# External Validation of a Novel Comprehensive Trifecta System in Predicting Oncologic and Functional Outcomes of Partial Nephrectomy: Results of a Multicentric Series

**DOI:** 10.3390/jcm11030796

**Published:** 2022-02-01

**Authors:** Umberto Anceschi, Rocco Simone Flammia, Daniele Mattevi, Antonio Tufano, Aldo Brassetti, Maria Consiglia Ferriero, Gabriele Tuderti, Leonardo Misuraca, Alfredo Maria Bove, Riccardo Mastroianni, Davide Marsiliani, Marco Puglisi, Tommaso Cai, Costantino Leonardo, Michele Gallucci, Gianni Malossini, Lorenzo Giuseppe Luciani, Giuseppe Simone

**Affiliations:** 1 Department of Urology, IRCCS “Regina Elena” National Cancer Institute, Via Elio Chianesi 53, 00144 Rome, Italy; aldo.brassetti@gmail.com (A.B.); marilia.ferriero@gmail.com (M.C.F.); gabriele.tuderti@gmail.com (G.T.); leonardo.misuraca@gmail.com (L.M.); alfredo.bove@ifo.gov.it (A.M.B.); riccardomastroianniroma@gmail.com (R.M.); puldet@gmail.com (G.S.); 2 Department of Maternal-Child and Urologic Sciences, Policlinico Umberto I, Sapienza University of Rome, Viale dell’Università 33, 00161 Rome, Italy; roccosimone92@gmail.com (R.S.F.); antonio.tufano@uniroma1.it (A.T.); costantino.leonardo@uniroma1.it (C.L.); michele.gallucci50@gmail.com (M.G.); 3 Department of Urology, APSS—Santa Chiara Regional Hospital, Largo Medaglie d’Oro 9, 38122 Trento, Italy; daniele.mattevi@apss.tn.it (D.M.); marco.puglisi@apss.tn.it (M.P.); ktommy@libero.it (T.C.); gianni.malossini@apss.tn.it (G.M.); lorenzo.luciani@apss.tn.it (L.G.L.); 4 Emergency Medicine Department, Presidio Ospedaliero G.B. Grassi, Via Gian Carlo Passeroni, 28, 00122 Ostia, Italy; mrsdvd@hotmail.it

**Keywords:** partial nephrectomy, trifecta, survival, end-stage renal disease (ESRD), ischemia

## Abstract

Background: To validate a novel trifecta for evaluating outcomes of partial nephrectomy (PN) on a multicentric dataset. Methods: Between 2007 and 2020, three renal cancer databases were queried for patients with solitary renal masses who underwent PN (n = 649). Trifecta was estimated for overall cohort and contributing centers. Overall survival (OS), cancer-specific survival (CSS) and end-stage renal disease (ESRD) probabilities were assessed by Kaplan–Meier. Cox regression was used to identify predictors of OS, CSS, ESRD. For all analyses, a *p* < 0.05 was considered significant. Results: At a median follow-up of 22.7 months (IQR 12.5–76.5) overall trifecta was 76.7% [Centre A; (n = 230; 68.6%), B (n = 68; 77.3%), C (n = 200; 88.4%); *p* = 0.001). On Kaplan–Meier, patients achieving trifecta exhibited higher OS (*p* = 0.024), higher CSS (*p* = 0.015) and lower ESRD rates (*p* = 0.024). On multivariable analysis, age (HR 1.04; 95% CI 1.01–1.08) and trifecta (HR 0.34; 95% CI 0.15–0.76) were independent predictors of OS while pT stage (HR 1.95; 95% CI 0.45–8.43) and trifecta (HR 0.33; 95% CI 0.16–0.67) were predictors of CSS (each *p* < 0.01). Preoperative CKD stage ≥ 3a (HR 13.1; 95% CI 4.07–42.6) and trifecta (HR 0.41; 95% CI 0.19–0.87) were independent predictors of ESRD (each *p* < 0.05). Conclusions: On external validation, trifecta was an independent predictor of all PN endpoints, regardless of hilar control and ischemia duration.

## 1. Introduction

Since its introduction, trifecta was conceived as a comprehensive way to optimize reporting of surgical outcomes and to objectively predict the risk of perioperative morbidity of partial nephrectomy (PN) [1]. To date, margin, ischemia, complications, score (MIC) and the original trifecta proposed by Khalifeh represent the most widely recognized reporting system for evaluating PN results [2,3]. They are commonly used for treatment planning, patient counselling and as a metric to improve reproducibility between PN series [4,5]. Nonetheless, to regulate surgical training, introduce technical innovations and describe outcomes with a simple and univocal terminology, the ability to predict both oncologic and functional endpoints of PN by these clinical tools remains an unmet need in the urological practice [6,7,8,9]. In recent years, there have been many attempts to modify the original trifecta definition, but several improvements in its predictive ability were offset by being a combination of variables more difficult to incorporate in a single scoring system [10,11].

Brassetti et al. have proposed a more comprehensive definition of the original trifecta version replacing the warm ischemia time (WIT) with perioperative estimated glomerular filtration rate (ΔGFR) variations, to also include clamp-less procedures [12]. Furthermore, in the same multicentric series, this novel trifecta consistently outperformed MIC in a rigorous head-to-head comparison showing a clear superiority in predicting clinically significant endpoints, namely overall survival and ESRD development probabilities [7].

However, external validation of this scoring system has not yet been published in the peer-reviewed environment, with the risk of an inconsistent pattern. Additionally, the recent introduction as the constraints in the estimated glomerular filtration rate (eGFR) variations threshold used for identifying acute kidney injury (AKI) may preclude the reproducibility of this trifecta on a larger scale [13]. To overcome these limitations, we sought to externally validate this clinical tool on a contemporary multicentric series of PNs and to test its predictive performances on all major endpoints of PN, namely overall survival (OS), cancer-specific survival (CSS) and newly onset of end-stage renal disease (ESRD).

## 2. Material and Methods

This retrospective multicentric study received an internal review board-approval at each contributing center. We retrieved data on 649 patients who underwent PN for cT1 renal masses between September 2007 and October 2020 at three participating institutions. Inclusion criteria were patients who were diagnosed with a single, organ-confined, contrast-enhancing, cT1a or cT1b non-metastatic renal mass. Indication to surgery was elective in all cases. Patients with tumors in solitary kidneys, or with multiple renal masses or with missing data were excluded from the present study. All patients were preoperatively evaluated with a computed tomography scan (CT) or magnetic resonance imaging (MRI).

Demographic, perioperative, pathological, oncologic and functional data were merged in a single, customized dataset. Evaluated preoperative clinical and demographic characteristics included age, gender, race, body mass index (BMI), American Society of Anesthesiologists (ASA) score, baseline estimated glomerular filtration rate (eGFR mL/min/1.73 m^2^), baseline chronic kidney disease (CKD) stage, clinical tumor size and RENAL nephrometry score. Perioperative variables included postoperative eGFR, ΔeGFR {([baseline eGFR- eGFR at discharge]/baseline eGFR)}, warm ischemia time (WIT), surgical approach (open, laparoscopic, robotic) clamping technique (yes/no), % perioperative complications, and surgical margin status (PSM). Oncologic outcomes included final histology, staging (according to TNM classification system), disease-free survival (DFS) and overall survival (OS). Functional outcomes consist of % newly onset of CKD stage 4.5 (ESRD) at last recorded follow-up.

Preoperative eGFR was calculated by the Chronic Kidney Disease Epidemiology Collaboration (CKD-EPI) [14] formula to determine baseline renal function. The ∆eGFR was estimated for evaluating the impact of the surgical procedure on renal function [12]. Baseline and postoperative CKD stage were assessed according to KDIGO International Guidelines [14]. Complications within 30 days after surgery were recorded and graded according to the Clavien–Dindo classification [15]. Major complications were categorized as Clavien Grade III or higher. Tumor size was selected by the largest dimension with the RENAL nephrometry scoring system to classify the complexity of tumor [16]. Pathological staging of RCC followed the TNM staging, including the nuclear grade by the Fuhrman system. Positive surgical margins (PSM) were defined by the presence of tumor cells at the level of parenchyma excision surface. Local recurrence was considered as cancer recurrence located either in previous tumor bed or in the Gerota’s fascia near to the enucleation area of partial nephrectomy (PN). All patients with PSM were followed up with thoraco-abdominal computed tomography scan every 6 months during the first year after treatment and every 12 months thereafter. All patients with negative margins status were followed up with thoraco-abdominal computed tomography scan at 6 months after surgery, then at 18 and 30 months and every 24 months thereafter, respectively.

Data were used to outline a binary variable for the achievement of trifecta (defined as the contemporary absence of positive surgical margins (PSM), major complications and ≤30% postoperative eGFR reduction) [12].

Primary endpoints of the study were to estimate trifecta achievement rate within the present study population and to identify predictors of overall survival (OS), cancer-specific survival (CSS) and end-stage renal disease (ESRD) by using univariable and multivariable Cox regression analysis. Descriptive analyses were used. Frequencies and proportions were reported for categorical variables while medians and interquartile ranges (IQRs) were reported for continuously coded variables. Differences between continuous variables were assessed with the one-way ANOVA test, while Pearson’s χ2 test was used for categorical data. OS, CSS and ESRD were computed by Kaplan–Meier curves and compared for trifecta achievement with the log-rank test, respectively

Secondary endpoints were to validate trifecta, relying on the original model described by Brassetti et al. [12]. Multivariable regression models were refitted, according to trifecta inclusion (full model) or exclusion (restricted model), within the original cohort (development cohort) and the current dataset (external validation cohort). AUC was computed for restricted and full models by using Heagerty’s method for censored survival data at 12 months for chronic kidney disease (CKD) stage 3b-5 upstaging (pCKD ≥ 3b) and at 60 months for overall mortality (OM), respectively. For all analyses, a two-sided *p* < 0.05 was considered significant. Statistical analysis was carried out using SPSS software v.26.0 (IBM Corp, Armonk, NY, USA).

## 3. Results

Descriptive statistics of the study population are shown in Table 1.

The median patient age was 64 years (IQR 55–72), while median clinical tumor size was 3.3 cm (IQR 2.4–4.5). Overall, 282 patients (43.4%) had a RENAL score of 4–6, 241 patients (37.1%) had a RENAL score of 7–9 and 126 patients (19.5%) had a RENAL score ≥10. Median preoperative eGFR was 83 (IQR 69–98.6). At baseline 232 patients had a CKD stage I (35.8%), 317 patients had a CKD stage II (48.6%), 68 patients had CKD stage 3a (10.5%) while 12 patients had a CKD stage IV–V (2%). There were significant differences in the distribution of case classifications among institutions. More in detail, in the A and C groups patients had significantly lower preoperative eGFR (*p* = 0.001) while in the B group patients showed significantly increased hypertension rates (*p* = 0.001), median clinical tumor size (*p* = 0.01). In the C group patients had higher cT stages (*p* = 0.001). With regard to the surgical approach used, in the A group there was a significantly higher number of both open (32.5%) and laparoscopic procedures (17%) (*p* = 0.001).

Perioperative and pathologic outcomes are summarized in Table 2.

Median WIT was 20 min (IQR 13–26). In the A group, 150 patients (35.4%) had a WIT > 20 min whereas in the B and C groups all patients underwent an off-clamp procedure. Positive surgical margins were observed in 52 patients (8%). The overall complications rate was 22.4%. The distribution by Clavien grade was 106 grade I-II complications (16.3%) and 40 grade III–V complications (6.1%). At final pathology, 74.5% of renal masses were malignant (n = 483) while 25.5% were benign tumors (n = 166). At a median follow-up of 22.7 months (IQR 12.5–76.5) 617 patients were alive (95%) while 42 patients (6.5%) developed a tumor recurrence.

In the A group the trifecta rate was 68.6%. More in detail, in the A group 126 patients (37.6%) achieved a WIT < 20 min with 283 patients (84.4%) showing a ΔeGFR ≤ 30%, 306 patients reported no major complications (91.3%). In the B group 68 patients (77.3%) achieved trifecta, with 82 patients (93.1%) revealing ΔegFR ≤ 30% and 83 patients (94.3%) reporting no major complications. In the C group 200 patients (88.4%) reached trifecta, with 206 patients (91.1%) reporting marginal ΔeGFR variations while the major complications rate was negligible (2.7%) (Table 3.)

With regard to functional outcomes, in the overall cohort 49 patients (7.5%) developed a CKD—stage 3b, and 33 patients ESRD (5%) (Table 3).

**Figure 1:**
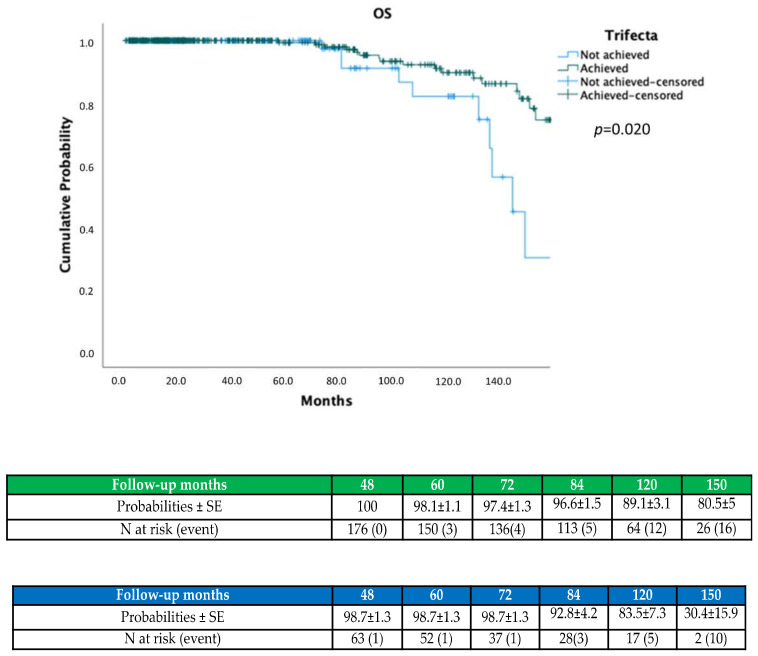
Kaplan–Meier showing OS probabilities according to trifecta achievement.

**Figure 2:**
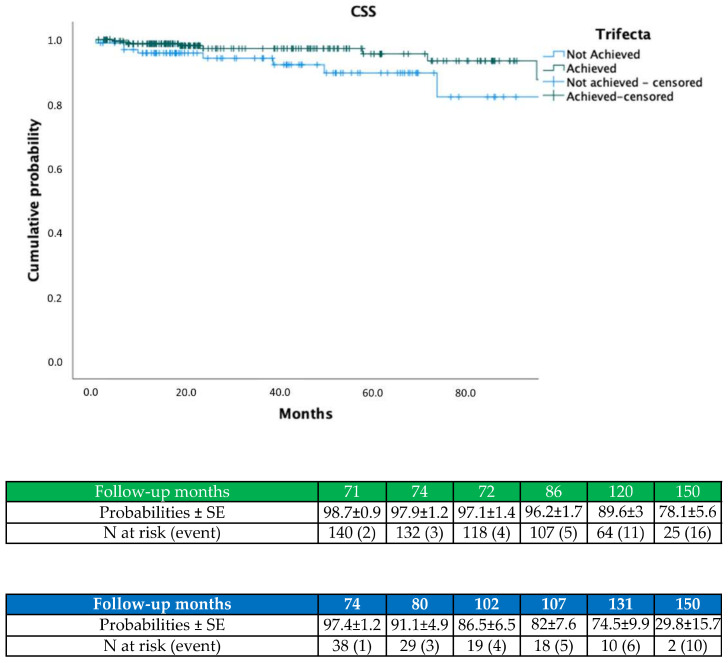
Kaplan–Meier showing CSS probabilities according to trifecta achievement.

**Figure 3:**
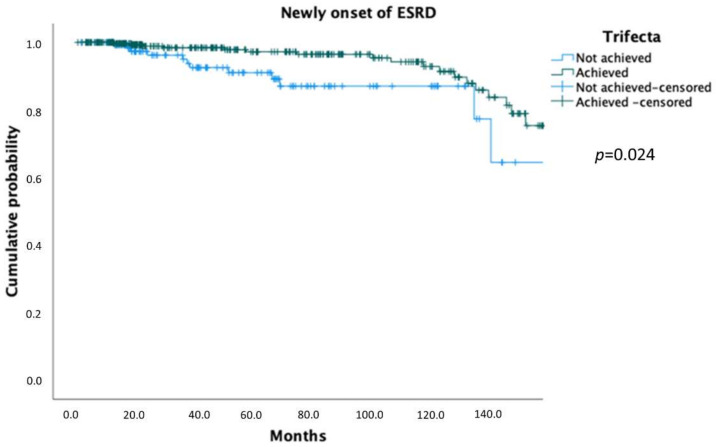

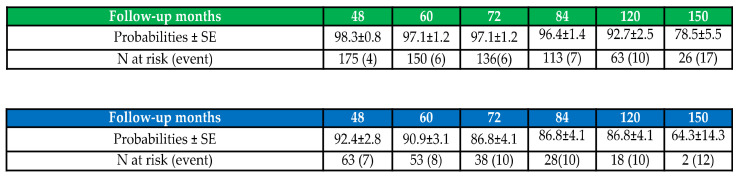
Kaplan–Meier showing ESRD probabilities according to trifecta achievement.

On Kaplan–Meier analysis, trifecta achievement predicted higher OS (*p* = 0.024; Figure 1), CSS (*p* = 0.015; Figure 2) and lower ESRD probabilities (*p* = 0.024; Figure 3).

On multivariable analysis, age at surgery (HR 1.04; 95% CI 1.01–1.08; *p* = 0.01) and trifecta (HR 0.34; 95% CI 0.15–0.76; *p* = 0.009) were independent predictors of OS (Table 4) while pT stage (HR 1.95; 95% CI 0.45–8.43; *p* = 0.006) and trifecta (HR 0.33; 95% CI 0.16–0.67; *p* = 0.002) were independent predictors of CSS (Table 5). When considering OS as outcome of interest, trifecta was an independent predictor either in the restricted or in the full model (Supplementary Table S1; each *p* < 0.03). In contrast, by replacing CSS with recurrence-free survival (RFS), trifecta was an independent predictor of RFS only in the current series (Supplementary Table S2; *p* = 0.008).

Preoperative CKD stage ≥ 3a (HR 13.1; 95% CI 4.07–42.6) and negative trifecta (HR 0.41; 95% CI 0.19–0.87) were independent predictors of newly onset of ESRD (each *p* < 0.05; Table 6). When the original Cox regression model was applied to the current series, replacing ESRD with pCKD ≥ 3b, negative trifecta was an independent predictor of significant renal deterioration (Supplementary Table S3; *p* = 0.034)

No difference in the AUC between the restricted and full model was reported in the development cohort when considering OM as the main endpoint. Regarding functional outcomes, the full model yielded a higher AUC than the restricted model within the development cohort. This difference was further confirmed when externally validated within the current series. (Table 7).

## 4. Discussion

The best “trifecta” for PN should consider reproducible and achievable criteria to predict major endpoints, independently of the surgical approach chosen and the hilar control used. To date, due to the complexity of surgical factors involved in PN, all trifecta systems shared an intrinsic limitation, considering warm ischemia time (WIT) as the univocal determinant of a composite outcome [7]. Since recent studies identified nephron mass preservation as the main key-factor for functional recovery after PN, the time threshold at which acute kidney injury (AKI) begins to occur during PN has been debated and there are still many ongoing controversies in this field [17,18,19]. Undoubtedly, off-clamp or selective clamping approaches to PN may obviate the burden of WIT from the trifecta equation, but such procedures are technically challenging and not universally yet adopted by the urological community [20,21,22]. Moreover, type and duration of ischemia plays a secondary role compared to the percentage of parenchymal mass preservation in the determination of renal function recovery. Consequently, novel trifecta definitions for PN must adapt to new perspectives.

The present study confirmed that perioperative eGFR variations (ΔeGFR) may represent a valid surrogate of WIT either as a clinical marker of acute kidney injury (AKI) or as predictor of major outcomes of PN. This parameter simplifies trifecta assignment in a more comprehensive fashion including clamp-less procedures, which be theoretically excluded using either current trifectas or MIC score. Since its description, our novel system proved to be not only comparable to standard MIC, but when included into a multivariable model it outperformed MIC in the predictive ability of OS, RFS and ESRD for robotic PN (RPN) [7]. However, the original series (n = 1807) evaluated only patients submitted exclusively to RPN in tertiary care centers. Consequently, trifecta achievement rate was higher (82%) and limited to a single surgical technique. Indeed, ischemia times was remarkably reduced (median 18.9 min) due to the large number of purely off-clamp procedures (36%), as the rate of perioperative morbidity reported (3%) [12]. Undoubtedly, all these factors may represent potential limitations for the reproducibility of our initial results.

In the current study, we also included open and laparoscopic PNs, considering a broader pattern of procedures compared to the original series. To date, no attempts of external validation of this novel system have been published. Furthermore, the present study investigated a large and contemporary cohort of PNs performed by surgeons out of learning curve to avoid a potential significant bias. The features of the series, in terms of surgical approach are representative of an historical and evolving scenario of PN. The increased number of perioperative grade I–II complications observed in the A group may represent a consequence of the higher number of patients with preoperative ASA III–IV score considered (A: 25.1% vs. B: 18.2% vs. C: 15.5%; *p* = 0.01). Similarly, considering wider follow-up timeframe in group A, a considerable bias represented by the evolution of the surgical technique as the totality of on-clamp procedures may be reflective of the lower trifecta achievement compared to other groups (A: 68.6% vs. B 77.3% vs. C: 88.4%; *p* = 0.001). Further disparities observed among centers in terms of ΔeGFR may be explained by the different impact of PN on a heterogeneous renal impairment (*p* = 0.09). In contrast to group A and C, patients in the B group showed a significative trend toward a negligible perioperative renal function worsening (*p* = 0.001).

At Cox regression analysis, the ability of trifecta to predict oncologic and functional outcomes was fairly absolute, both in pairwise univariable and multivariable fashion, for all endpoints considered (Table 4, Table 5 and Table 6). By duplicating the original Cox regression models described by Brassetti et al. in the current population, the addition of Trifecta increased the accuracy of multivariable models predicting both functional and oncologic outcomes in the external validation (Supplementary Tables S1–S3 and Table 7). It must be underlined that restricted and full models predicting CKD progression showed optimal accuracy (>0.90) in the external validation. Conversely, both restricted and full models predicting OM showed low accuracy (<0.60) in the external validation. It can be postulated that differences between development and current study cohort, as well as low generalizability of the original models predicting OM, built by Brassetti et al., have accounted for the low accuracy reported for OM in this external validation.

In view of all these data, it seems that the clinical utility of our trifecta is not limited only to an easier standardization of perioperative outcomes reporting but also predictive of major endpoints of PN at a mid-term follow-up (median 22.7 months). More in detail, in this series trifecta (*p* = 0.002) and pT stage (*p* = 0.006) were independent predictors of CSS while in a previous study, trifecta achievement significantly predicted recurrence-free survival (RFS) only at univariable analysis [7]. This finding may be explained by the different distribution of patients among groups in terms of cT stage (cT ≥2a: Group A 4.1% vs. Group B 9% vs. Group 8.5%) and the higher trend of PSM rates observed in A and B groups (A: 12.5% vs. B: 11.3% vs. C: 0%; *p* = 0.001), respectively. Moreover, the use of CSS instead of RFS as oncologic endpoint as the wider timeframe considered in the A group (range 43.9–118.4 months) could potentially have captured sporadic or metachronous recurrences and may not necessarily reflect a matter of surgical quality.

With regard to functional outcomes, our study showed interesting findings. At univariable and multivariable Cox regression analysis, neither WIT, hilar control or RENAL score were predictive of newly onset ESRD at an extended follow-up (each *p* < 0.5). Conversely, our data enhanced the role of pre-existing renal impairment and major comorbidities such as diabetes as key determinants of unavoidable functional decline after PN independently of tumor complexity, and the utility of trifecta achievement as an overarching indicator of mid-term kidney functional maintenance (Table 6) [23,24].

Our study is not devoid of limitations. Undoubtedly, we considered a limited series with small renal masses which may limit the generalizability of our validation to more complex populations. Moreover, since robotic surgery accounted for 74.1% of procedures and creatinine levels usually peak at day 3–4 in the postoperative setting, an early discharge rate may have indirectly affected renal function estimation in our cohort. Furthermore, the retrospective determination of trifecta is subject to associated biases due to the wide timeframe of the series considered and different perioperative factors present in our dataset, mainly the surgical approach used and the hilar control technique. Additionally, since the loss of renal function could be significantly affected by different preoperative and intraoperative factors, we were unable to adjust regression models for the severity of comorbidities, tumor resection strategies, and a rigorous measurement of parenchymal mass preserved after surgery in this multicentric database. Finally, the arbitrary threshold used for identifying a significative post-operative renal impairment (ΔegFR ≥ 30) instead of WIT, as the lack of parenchymal adjustment for WIT remain undeniable aspects to consider in this population.

Nonetheless, this validation study duplicated the promising outcomes showed by our trifecta in the original series. In this multicentric dataset, trifecta achievement independently predicted all key major outcomes of PN. Validation of this novel trifecta represents a step forward in considering ΔegFR ≥ 30 as a reasonable and reproducible threshold for identifying clinically significant AKI instead of WIT during PN.

## 5. Conclusions

We externally validated a novel trifecta system for predicting major oncologic and functional outcomes of PN for small renal masses at mid-term follow-up. This new and easy clinical tool confirmed to be a comprehensive marker of surgical quality and a safe method for evaluating oncologic outcomes and development of ESRD at an intermediate timeframe. This external validation might aid in promoting the use of this predictive tool for reliably estimating survival and functional outcomes after PN, independently of the surgical approach considered.

## Figures and Tables

**Table 1:** Baseline and demographic data.

Variable	Overall Cohort (n = 649)	Centre A (n = 335)	Centre B (n = 88)	Centre C (n = 226)	*p*
Age (yrs, median, IQR)	64 (55–72)	64 (55–72)	63 (54.2–71)	62.5 (54–72.5)	0.710
Gender (n,%) Male Female	416 (64%) 233 (36%)	216 (64.5%) 119 (35.5%)	52 (59.1%) 36 (40.9%)	148 (65.5%) 78 (34.5%)	0.557
ASA score (n,%) 1–2 3–4	514 (79.1%) 135 (20.9%)	251 (74.9%) 84 (25.1%)	72 (81.8%) 16 (18.2%)	191 (84.5%) 35 (15.5%)	0.01
Diabetes (n,%)	60 (9.2%)	32 (9.5%)	14 (15.9%)	14 (6.2%)	0.02
Hypertension (n,%)	197 (30.3%)	122 (36.4%)	46 (52.2%)	29 (12.8%)	0.001
Surgical approach (n,%) Open Laparoscopic Robotic	109 (16.7%) 59 (9.2%) 481 (74.1%)	109 (32.5%) 57 (17%) 169 (50.4%)	- 2 (2.3%) 86 (97.7%)	- - 226 (100%)	0.001
Clinical tumor size (cm, median, IQR)	3.3 (2.4–4.5)	3.2 (2.2–4.2)	3.9(2.6–5)	3.2 (2.5–4.5)	0.06
RENAL score (n,%) 4–6 7–9 10–12	282 (43.4%) 241 (37.1%) 126 (19.5%)	143 (42.6%) 120 (35.8%) 72 (21.7%)	31 (34%) 42 (47.7%) 15 (17%)	108 (47.8%) 79 (35%) 39 (17.2%)	0.124
Preoperative eGFR (mL/min/1.73 m^2^, median, IQR)	83 (69–98.6)	82.2 (69.3–95.3)	90.2 (67–118)	81.5 (70–97.7)	0.001
Preoperative cT stage (n,%) 1a 1b 2a 2b	440 (67.8%) 168 (25.9%) 35 (5.4%) 6 (0.9%)	248 (74%) 73 (21.7%) 14 (4.1%) -	49 (55.6%) 31 (35.2%) 4 (4.5%) 4 (4.5%)	143 (63.2%) 64 (28.3%) 17 (7.5%) 2 (1%)	0.001
Preoperative CKD stage (n,%) 1 2 3a 3b 4 5	232 (35.8%) 317 (48.6%) 68 (10.5%) 20 (3.1%) 10 (1.6%) 2 (0.4%)	120 (35.8%) 165 (49.2%) 34 (10.1%) 8 (2.38%) 8 (2.38%) -	45 (51.1%) 34 (38.6%) 7 (7.9%) 2 (2.2%) -	67 (29.7%) 118 (52.3%) 27 (12%) 10 (4.4%) 2 (0.8%) 2 (0.8%)	0.09

**Table 2:** Perioperative and pathologic outcomes.

Variable	Overall Cohort (n = 649)	Centre A (n = 335)	Centre B (n = 88)	Centre C (n = 226)	*p*
PSM (n,%)	52 (8%)	42 (12.5%)	10 (11.3%)	0 (%)	0.001
Hystology type (n,%) Benign Malignant	166 (25.5%) 483 (74.5%)	71 (21.2%) 264 (78.8%)	22 (25%) 66 (75%)	73 (32.3%) 153 (67.7%)	0.01
pT stage (n,%) 1a 1b 2a 2b 3a 3b	388 (59.8%) 176 (27.2%) 55 (8.5%) 18 (2.8%) 9 (1.3%) 3 (0.4%)	232 (69.2%) 67 (20%) 31 (9.2%) - 2 (0.6%) 3 (0.9%)	41 (46.5%) 29 (32.9%) 10 (11.3%) 5 (5.6%) 3 (3.4%) -	115 (50.8%) 80 (35.4%) 14 (6.2%) 13 (5.8%) 4 (1.8%) -	0.001
Overall perioperative complications (any, n,%)	146 (22.4%)	113 (33.7%)	10 (11.3%)	23 (10.2%)	0.001
Postoperative Clavien Grade (n,%) 1–2 3–5	106 (16.3%) 40 (6.1%)	84 (25%) 29 (8.65%)	5 (5.68%) 5 (5.68%)	17 (7.5%) 6 (2.6%)	0.001 0.01
WIT (median, min, IQR)	0 (0–20)	20 (13–26)	-	-	0.001
Postoperative eGFR (mL/min/1.73 m^2^ median, IQR)	80 (62.2–100.9)	80.2 (60.9–103.4)	91.7 (68.3–130–7)	77 (58.2–91)	0.001
ΔeGFR (mL/min/1.73 m^2^ median, IQR)	3.3 (−14.5; +18.06)	0 (−19; +19)	−4.5 (−17.8; +7.6)	8.38 (0–19)	0.001

**Table 3:** Functional outcomes.

Variable	Overall Cohort (n = 649)	Centre A (n = 335)	Centre B (n = 88)	Centre C (n = 226)	*p*
Follow-up (months, median, IQR)	22.7 (12.5–76.5)	73.4 (43.9–118.4)	16.4 (13.8–19.8)	9.2 (4.2–15.8)	0.001
OS (n,%)	617 (95%)	304 (90.7%)	88 (100%)	225 (99.6%)	0.001
CSS (n,%)	607 (93.5%)	299 (89.2%)	84 (95.5%)	224 (99.1%)	0.001
Recurrence (n,%) * Local Renal	35 (7.2%) 7 (1.4%)	30 (6.2%) 6 (1.2%)	3 (0.6%) 1 (0.2%)	2 (0.4%) -	0.001
eGFR at last follow-up (mL/min/1.73 m^2^, median, IQR)	63.2(49.2–76.1)	57.3 (45.4–71.4)	69.2 (59.6–85.6)	70.1 (59.6–85.8)	0.001
Newly onset CKD 3b (n,%)	49 (7.5%)	43 (12.8%)	5 (5.7%)	3 (1.3%)	0.004
Newly onset ESRD (n,%)	33 (5%)	28 (8.4%)	4 (4.5%)	1 (0.4%)	0.02
CKD stages at last follow-up (n,%) 1 2 3a 3b 4 5	102 (15.8%) 301 (46.4%) 142 (21.8%) 64 (9.8%) 26 (4.1%) 14 (2.1%)	18 (5.4%) 141 (42.1%) 100 (29.9%) 45 (13.4%) 20 (6%) 11 (3.3%)	17 (19.3%) 48 (54.5%) 13 (14.8%) 6 (6.8%) 3 (3.4%) 1 (1.1%)	67 (29.7%) 112 (49.6%) 29 (12.9%) 13 (5.7%) 3 (1.3%) 2 (0.8%)	0.001
Trifecta (n,%) ΔeGFR ≤ 30 Negative surgical margins No Clavien ≥ 3	498 (76.7%) 571 (87.9%) 597 (93.2%) 609 (93.8%)	230 (68.6%) 283 (84.4%) 293 (87.4%) 306 (91.3%)	68 (77.3%) 82 (93.1%) 78 (88.6%) 83 (94.3%)	200 (88.4%) 206 (91.1%) 226 (100%) 220 (97.3%)	0.001

* Recurrence rate was calculated only for malignant lesions (n = 483).

**Table 4:** Cox regression analysis to identify predictors of OS.

Variable	Univariable Analysis				Multivariable Analysis			
	HR	95.0% CI			HR	95.0% CI		
		Lower	Higher	*p* Value		Lower	Higher	*p* Value
Age	1.04	1.01	1.07	0.02	1.04	1.01	1.08	0.01
Gender	0.80	0.36	1.75	0.578	-	-	-	-
Diabetes	1.19	0.36	3.96	0.769	-	-	-	-
Hypertension	1.13	0.54	2.35	0.739	-	-	-	-
ASA score 1–2 3–4	0.89	0.34	2.36	0.829	-	-	-	-
pT stage	0.85	0.31	2.29	0.756	-	-	-	-
RENAL (cat) 4–6 vs. 7–9 4–6 vs. 10–12	1.06 1.55	0.45 0.51	2.49 4.75	0.884 0.435	-	-	-	-
Preoperative CKD stage	0.52	0.11	2.38	0.403	-	-	-	-
Trifecta	0.42	0.19	0.91	0.029	0.34	0.15	0.76	0.009

**Table 5:** Cox regression analysis to identify predictors of CSS *.

Variable	Univariable Analysis				Multivariable Analysis			
	HR	95.0% CI			HR	95.0% CI		
		Lower	Higher	*p* Value		Lower	Higher	*p* Value
Age	1.01	0.97	1.04	0.715	-	-	-	-
Gender	0.77	0.35	1.70	0.515	-	-	-	-
Diabetes	0.47	0.06	3.48	0.459	-	-	-	-
Hypertension	1.14	0.52	2.54	0.740	-	-	-	-
ASA score 1–2 3–4	1.59	0.55	4.61	0.391	-	-	-	-
RENAL (cat) 4–6 vs. 7–9 4–6 vs. 10–12	1.90 2.19	0.43 0.47	8.37 10.09	0.396 0.316	-	-	-	-
pT stage	1.15	0.82	1.60	0.429	1.16	0.83	1.62	0.339
Preoperative CKD stage	1.12	0.76	1.66	0.569	-	-	-	-
Trifecta	0.36	0.17	0.77	0.008	0.36	0.17	0.77	0.008

* Adjusted for malignant lesions only.

**Table 6:** Cox regression analysis to identify predictors of ESRD.

Variable	Univariable Analysis				Multivariable Analysis			
	HR	95.0% CI			HR	95.0% CI		
		Lower	Higher	*p* Value		Lower	Higher	*p* Value
Age	1.01	0.97	1.04	0.580	-	-	-	-
Gender	1.13	0.56	2.28	0.721	-	-	-	-
Diabetes	3.14	1.40	7.02	0.005	1.29	0.50	3.29	0.595
Hypertension	1.73	0.87	3.44	0.116	-	-	-	-
ASA score 1–2 3–4	1.31	0.58	2.96	0.503	-	-	-	-
RENAL (cat) 4–6 vs. 7–9 4–6 vs. 10–12	1.23 1.30	0.57 0.42	2.68 4.01	0.589 0.639	-	-	-	-
Hilar control	1.52	0.61	3.82	0.363	-	-	-	-
Warm Ischemia Time	1.01	0.98	1.05	0.339	-	-	-	-
Preoperative CKD stage CKD 3a vs. CKD 1–2 CKD 3a vs. CKD 3b	0.04 0.11	0.01 0.02	0.13 0.44	<0.001 0.002	4.55 13.1	1.51 4.07	13.7 42.6	0.007 <0.001
Trifecta	0.47	0.22	0.98	0.045	0.41	0.19	0.87	0.02

**Table 7:** AUC calculated at both 12 months for pCKD ≥ 3b and 60 months for overall mortality (OM) within original series [12] and external validation cohort using Heagerty’s method for censored survival data.

		Development Cohort	External Validation Cohort
pCKD ≥ 3b	AUC restricted model	0.915	0.907
	AUC full model *	0.920	0.946
OM	AUC restricted model	0.858	0.503
	AUC full model *	0.859	0.593

* This matched exactly the results from Brassetti et al. [12].

## Data Availability

The data presented in this study are available on request from the corresponding author. The data are not publicly available due to privacy restrictions.

## Supplementary Materials

The following supporting information can be downloaded at: https://www.mdpi.com/article/10.3390/jcm11030796/s1, Table S1: Cox regression predicting overall mortality, in the original series [12] and in the external validation cohort; Table S2: Cox regression predicting cancer-specific survival, in the original series [12] and in the external validation cohort; Table S3: Cox regression predicting newly onset of CKD3b, in the original series [12] and in the external validation cohort.
