# Supplementary material for: External Validation of a Novel Comprehensive Trifecta System in Predicting Oncologic and Functional Outcomes of Partial Nephrectomy: Results of a Multicentric Series
Source: J Clin Med. 2022 Feb 1;11(3):796. doi: 10.3390/jcm11030796 (PMC8837057; doi:10.3390/jcm11030796)
Supplement: Supplementary file 1 [file jcm-11-00796-s001.zip › jcm-1543221-supplementary.pdf]

Supplementary Table S1. Cox regression predicting overall mortality, in the original series [12] and in the external validation cohort*.

Variable	Univariable analysis				Multivariable analysis			
	HR	95.0% CI			HR	95.0% CI		
		Lower	Higher	p value		Lower	Higher	p value
**Age**	1.04	1.01	1.07	0.02	1.04	1.01	1.08	0.01
**Gender**	0.80	0.36	1.75	0.578	-	-	-	-
**Diabetes**	1.19	0.36	3.96	0.769	-	-	-	-
**Hypertension**	1.13	0.54	2.35	0.739	-	-	-	-
**ASA score**<br>**1-2**<br>**3-4**	0.89	0.34	2.36	0.829	-	-	-	-
**pT stage**	0.85	0.31	2.29	0.756	-	-	-	-
**RENAL (cat)**					-	-	-	-
**4-6 vs 7-9**	1.06	0.45	2.49	0.884				
**4-6 vs 10-12**	1.55	0.51	4.75	0.435				
**Preoperative CKD stage**	0.52	0.11	2.38	0.403	-	-	-	-
**Trifecta**	0.42	0.19	0.91	0.029	0.34	0.15	0.76	0.009

Supplementary Table S2. Cox regression predicting CSS survival, in the original series [12] and in the external validation cohort*.

	Multivariable Cox regression model							
	Development cohort *				Externally validation cohort			
	Restricted model		Full model		Restricted model		Full model	
**Variable**	**HR (95%IC)**	**p**	**HR (95%IC)**	**p**	**HR (95%IC)**	**p**	**HR (95%IC)**	**p**
**Trifecta (yes vs no)**	-	-	0.79 (0.35-1.79)	0.572	-	-	0.41 (0.35-1.19)	0.008
**WIT**	1.08 (0.99-1.17)	0.071	1.07 (0.99-1.16)	0.081	1.05 (1.01-1.09)	0.028	1.04 (0.99-1.16)	0.039
**Tumor size**	0.97 (0.93-1.01)	0.095	0.97 (0.93-1.01)	0.107	1.00 (0.83-1.22)	0.962	1.00 (0.93-1.01)	0.996
**Preop. eGFR**	0.99 (0.98-1.01)	0.353	0.99 (0.98-1.01)	0.353	1.00 (0.99-1.02)	0.777	1.00 (0.98-1.01)	0.922
**RENAL**	1.42 (1.19-1.70)	<0.001	1.42 (1.19-1.70)	<0.001	1.02 (0.87-1.20)	0.813	1.04 (1.19-1.70)	0.634
**Off-clamp**	5.90 (1.1 – 32.8)	0.043	5.69 (1.02-31.7)	0.048	2.86 (0.88-9.31)	0.081	2.73 (1.02-31.7)	0.094

** This matched exactly the results from Brassetti et al [12]. Adjusted for malignant lesions only.*

Supplementary Table S3. Cox regression predicting newly onset of CKD≥3b, in the original series [12] and in the external validation cohort*.

	Multivariable Cox regression model							
	Development cohort *				Externally validation cohort			
	Restricted model		Full model		Restricted model		Full model	
Variable	HR 95%IC	p-value	HR 95%IC	p	HR 95%IC	p-value	HR 95%IC	p
**Trifecta (yes vs no)**	-	-	0.35 (0.19-0.62)	<0.001	-	-	0.59 (0.36-0.96)	0.034
**WIT**	1.02 (0.99-1.05)	0.081	1.01 (0.98-1.03)	0.947	1.04 (1.01-1.07)	0.021	1.03 (1.01-1.07)	0.028
**Preop. eGFR**	0.95 (0.94-0.97)	<0.001	0.95 (0.94-0.96)	<0.001	0.99 (0.98-0.99)	0.013	0.99 (0.98-0.99)	0.013
**Off-clamp**	1.11 (0.44- 2.80)	0.833	0.82 (0.32-2.15)	0.690	1.63 (0.72-3.70)	0.246	1.56 (0.70-3.63)	0.266
**Age**	1.01 (0.99-1.04)	0.250	1.01 (0.99-1.04)	0.398	1.01 (0.98-1.02)	0.904	1.01 (0.98-1.03)	0.745
**BMI**	1.01 (0.97-1.06)	0.529	1.01 (0.965-1.06)	0.697	1.04 (0.98-1.09)	0.191	1.03 (0.98-1.09)	0.214
**ASA**	1.40 (0.95-2.05)	0.087	1.43 (0.99-2.13)	0.079	1.19 (0.82-1.73)	0.355	1.21 (0.83-1.74)	0.320
**RENAL**	1.18 (1.09-1.37)	0.028	1.22 (1.05-1.41)	0.009	1.07 (0.96-1.19)	0.211	1.09 (0.97-1.21)	0.140

** This matched exactly the results from Brassetti et al [12].*
